# CRISPR-Generated Animal Models of Duchenne Muscular Dystrophy

**DOI:** 10.3390/genes11030342

**Published:** 2020-03-24

**Authors:** Kenji Rowel Q. Lim, Quynh Nguyen, Kasia Dzierlega, Yiqing Huang, Toshifumi Yokota

**Affiliations:** 1 Department of Medical Genetics, Faculty of Medicine and Dentistry, University of Alberta, Edmonton, AB T6G 2H7, Canada; kenjirow@ualberta.ca (K.R.Q.L.); nguyenth@ualberta.ca (Q.N.); dzierleg@ualberta.ca (K.D.); yiqing11@ualberta.ca (Y.H.); 2 The Friends of Garrett Cumming Research & Muscular Dystrophy Canada, HM Toupin Neurological Science Research Chair, Edmonton, AB T6G 2H7, Canada

**Keywords:** Duchenne muscular dystrophy, CRISPR, animal models, in vivo testing, dystrophin, mutant generation

## Abstract

Duchenne muscular dystrophy (DMD) is a fatal X-linked recessive neuromuscular disorder most commonly caused by mutations disrupting the reading frame of the dystrophin (*DMD*) gene. *DMD* codes for dystrophin, which is critical for maintaining the integrity of muscle cell membranes. Without dystrophin, muscle cells receive heightened mechanical stress, becoming more susceptible to damage. An active body of research continues to explore therapeutic treatments for DMD as well as to further our understanding of the disease. These efforts rely on having reliable animal models that accurately recapitulate disease presentation in humans. While current animal models of DMD have served this purpose well to some extent, each has its own limitations. To help overcome this, clustered regularly interspaced short palindromic repeat (CRISPR)-based technology has been extremely useful in creating novel animal models for DMD. This review focuses on animal models developed for DMD that have been created using CRISPR, their advantages and disadvantages as well as their applications in the DMD field.

## 1. Introduction

Duchenne muscular dystrophy (DMD) is a fatal X-linked recessive neuromuscular disorder characterized by progressive muscle degeneration and weakness [1]. DMD affects 1 in 3500–5000 males born worldwide, making it the most common inherited neuromuscular disorder [2,3]. DMD is caused by loss-of-function mutations in the dystrophin (*DMD*) gene which codes for a cytoskeletal protein called dystrophin [1,4]. Dystrophin functions via the dystrophin glycoprotein complex (DGC) to link the actin cytoskeleton of muscle cells to the extracellular matrix, providing mechanical support to myofibers during cycles of contraction and relaxation [5,6,7]. In the absence of dystrophin, myofibers become susceptible to tearing and fragmentation, resulting in muscle degeneration. Patients with DMD often present their first symptoms as early as 3–5 years of age [8,9]. The disease progresses rapidly, with muscle weakness and wasting observed initially in the proximal muscles, spreading distally. Patients experience multiple organ system dysfunction, and often lose their mobility by the age of 12 years [8,9]. Death usually occurs in the second or third decade of life due to respiratory and/or cardiac complications [10].

Currently, there is no cure for DMD. Animal models are available to investigate potential treatments for DMD, as well as to better understand its pathogenesis [11,12,13,14]. The majority of pre-clinical studies for candidate DMD therapies have been carried out in murine models, mostly the *mdx* mouse that carries a nonsense point mutation in *Dmd* exon 23 [15]. Genetic therapies that have been tested in these mouse models include micro/mini-dystrophin delivery, dystrophin transcript-level correction via stop codon read-through or exon skipping, and dystrophin gene repair via genome editing [16,17]. The first two strategies have moved on to clinical trials, with exon skipping being particularly successful. In fact, the first US FDA-approved therapeutic for DMD is an exon 51-skipping drug called eteplirsen (Exondys 51, Sarepta) [18]; this was followed by approval of the exon 53-skipping drug golodirsen (Vyondys 53, Sarepta) [19] approximately three years later. The use of canine models has been gaining ground as well, with a gene-editing therapy having been recently tested on dogs with an out-of-frame *Dystrophin* exon 50 deletion [20]. While these models have definitely advanced our efforts to treat DMD, they have their limitations. For instance, murine models do not faithfully recapitulate the phenotypes found in DMD patients [12,15,21]. The *mdx* mouse shows much milder phenotypes, with no severe cardiac involvement; lacks a pronounced effect of the disease on survival; has regenerative and compensatory mechanisms in response to dystrophin loss; and represents a single mutation out of the possible thousands found in patients [22,23,24,25,26]. Canine models exhibit phenotypes closer to patients. However, disease severity is extremely variable among littermates [27,28] and they are also limited by the number of available mutations for study. There is thus a need to develop new animal models to better represent both the phenotype and mutational spectrum of DMD.

Clustered regularly interspaced short palindromic repeat (CRISPR)-based technology has been recently adapted to create animal models for DMD, among other diseases [29]. CRISPR is a genome-editing tool adapted from the prokaryotic adaptive immune system [30]. When used with nucleases such as CRISPR-associated protein 9 (Cas9), CRISPR allows for the precise editing of virtually any target gene [31,32]. With such capabilities, CRISPR has revolutionized the DMD field, not only as an alternative therapeutic strategy for the disorder but also in providing new in vitro and in vivo DMD models [33]. Here, we aim to comprehensively review the DMD animal models that have been created so far using CRISPR. We describe each of these models and their applications, as well as their advantages and disadvantages. We conclude with some challenges and future prospects on the use of these in vivo models in the field.

## 2. Overview of Genome Editing by CRISPR

The discovery of CRISPR as an antiviral defense system in bacteria introduced large strides in genetic manipulation and molecular biology research [34]. Simply put, the CRISPR system is an RNA-guided endonuclease capable of introducing targeted mutations in the genome [35,36]. CRISPR specificity is achieved by designing guide RNAs (gRNAs) with sequence complementarity to the target locus. In the most common configuration of CRISPR presently used, these gRNAs are co-administered with the Cas gene or enzyme of choice, e.g., Cas9, to induce DNA double-strand breaks (DSBs) [35]. DNA DSBs are repaired either by non-homologous end joining (NHEJ) or homology-directed repair (HDR) for random or highly specific editing, respectively. The identification of target sequences by the gRNA–Cas complex is mediated by the recognition of protospacer adjacent motifs (PAMs). These conserved sequences must be located downstream of target sites, ensuring the precise nature of CRISPR/Cas activity [37].

Many CRISPR systems have been identified and adapted from different bacterial species, increasing the versatility of genome editing with the approach. For instance, numerous Cas9 variants have been discovered other than the widely used SpCas9 from *Streptococcus pyogenes* (Table 1). Not only does this expand the number of potential target sites for CRISPR-mediated genome editing, but also offers certain advantages over SpCas9, e.g., in terms of size and ease of packaging into viral vectors. Cas enzymes of other types and classes have also been studied, further enriching the capabilities of CRISPR editing. For instance, there is Cpf1, a class-2, type V CRISPR endonuclease that produces sticky ends after DNA DSB induction, requires shorter gRNAs, and has stricter PAM site requirements compared to Cas9 (class-2, type II).

Various studies have demonstrated the capacity of CRISPR to treat genetic diseases including cystic fibrosis, blood disorders, Huntington’s disease, cardiovascular disease and DMD [50,51,52,53,54]. However, CRISPR has notably also allowed for the construction of novel animal disease models [55]. The low cost and simplicity of the CRISPR/Cas system, along with its ability to efficiently create mutations at any genomic location in virtually any species, prove to be advantageous in the development of animal models for human diseases [56]. By introducing multiple gRNAs, CRISPR can target multiple loci simultaneously [57]. Successful generation of multiple mutations in animals was demonstrated by Wang et al. (2013), where they simultaneously introduced mutations in eight alleles [58]. The use of CRISPR also shortens the amount of time it takes to create a transgenic animal. Whereas the classical vector-mediated homologous recombination approach typically takes 1–2 years on average to generate transgenic mice, some applications of CRISPR can reduce this to 1–2 months [59,60]. All these improvements are made possible by the small number of reagents required (as little as two reagents—Cas9 mRNA/protein and gRNA—have to be microinjected into zygotes), the ease of reagent delivery (a direct microinjection), the high efficiency associated with CRISPR, and the bypassing of complex steps that characterized earlier methods of transgenic animal creation, such as the need for using embryonic stem cells and extensive breeding strategies [58,61,62]. Animal model development by CRISPR, therefore, presents numerous advantages over traditional methods, allowing for the study of disease mechanisms and potential treatments. In an earlier paper, we have extensively reviewed the utility of CRISPR in developing genome-editing therapies for DMD [33]. Now, we review how CRISPR has been used to generate animal models of DMD.

## 3. DMD Animal Models Generated by CRISPR-Mediated Genome Editing

**Figure 1:**
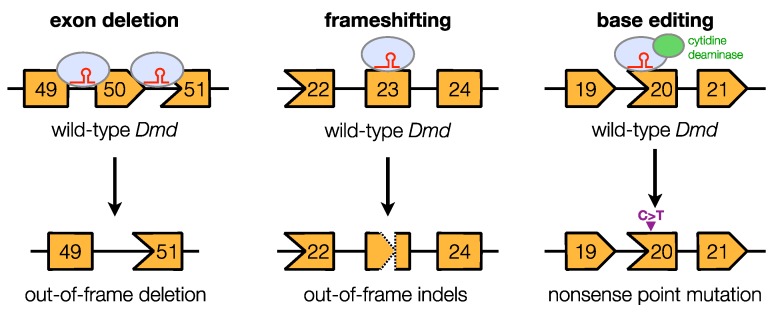
Clustered regularly interspaced short palindromic repeat (CRISPR)-mediated genome editing for Duchenne muscular dystrophy animal model development. An overview of the different genome-editing approaches used to create dystrophin gene mutations for animal model creation is shown. Boxes, exons; blue circles with red line structures, gRNA–Cas complexes.

Table 2 summarizes the CRISPR-generated DMD animal models discussed in this section, and Table 3 provides a general overview of each of their advantages and disadvantages. These models were generated through a variety of approaches, including the deletion of one or more exons, as well as the introduction of frameshift or point mutations within exons (Figure 1).

### 3.1. Mouse

To overcome the challenges mentioned earlier on use of the *mdx* mouse, particularly with regard to its mild phenotype, numerous groups have developed DMD mouse models of their own by either crossing the *mdx* mutation to other strains of mice, using chemical mutagenesis to generate new mutations in other regions of the *Dmd* gene, or by generating targeted disruptions or a total knockout of the gene [24]. For instance, so-called “double knockout” or dKO mice deficient in both dystrophin and its homolog utrophin (*mdx*; *utrn*^-/-^) have been developed, which show much more severe phenotypes than the regular *mdx* mouse, including decreased cardiac function and survival [26,70]. Continuing these efforts, CRISPR offers more versatility, efficiency, and simplicity in the creation of new DMD mouse models. The ability of CRISPR to expand the number of mutations that can be modelled in vivo is also especially helpful for the development of personalized therapies such as exon skipping or genome editing, which requires specific mutation backgrounds for testing.

DMD mouse models carrying deletions, frameshifting mutations, a point mutation, and a mutant version of the human *DMD* gene have been generated through CRISPR. Amoasii et al. (2017) created a model carrying an out-of-frame deletion of *Dmd* exon 50 [64]. Male ΔEx50 mice showed an overall absence of dystrophin in skeletal and cardiac muscles through Western blot and immunostaining, with few revertant fibers (RFs). Skeletal muscle necrosis was observed by histology at 3 weeks of age. At 2 months, elevated serum creatine kinase (CK) levels, a significant increase in the percentage of centrally nucleated fibers (CNFs), and a significant decrease in forelimb grip strength by ~50% were found in male ΔEx50 mice compared to wild-type controls. Symptoms were similar to those in *mdx* mice. Two years later, the same group created a variant of this model with a luciferase construct at the 3′ end of the mutant ΔEx50 *Dmd* gene [69]. In these ΔEx50-Dmd-Luc mice, an autocatalytic reaction releases luciferase from the rest of the dystrophin protein soon after translation. Aside from having similar dystrophic phenotypes as the ΔEx50 mice, this model allows for the non-invasive monitoring of dystrophin rescue, which is useful for treatment time-course studies or better understanding the pharmacological properties of DMD therapeutics. Both models have been used for testing CRISPR therapies aiming to reframe or skip exon 51 [64,69].

The ΔEx44 mouse by Min et al. (2019) is another DMD model made by CRISPR, this one having exon 44 removed instead [67]. Dystrophin was not detected through Western blot and immunostaining in the examined skeletal muscles as well as the heart. By 4 weeks of age, typical dystrophic features were observed by histology in male ΔEx44 mice, including a significant increase in serum CK levels compared to wild-type controls. Specific force of the extensor digitorum longus (EDL) and forelimb grip strength were both significantly reduced by ~50% versus wild-type. Along with the ΔEx50 mouse, the ΔEx44 model can be used for testing exon-skipping or genome-editing therapies targeting the distal exon 43–55 mutation hotspot of the *Dmd* gene. As for testing therapies for the proximal exon 1–22 mutation hotspot, the ΔEx8-34 (or DMD^del8-34^) mouse model made by Egorova et al. (2019) could be used. This model had a similar phenotype as the *mdx* mice, with an absence of dystrophin in the tibialis anterior (TA) as well as significant decreases in muscle function, significant increases in serum CK levels, and significant dystrophic histopathology as early as 2–3 months compared to wild-type [71]. Moreover, the ΔEx8-34 model has the largest deletion ever generated in vivo of the *Dmd* gene through CRISPR/Cas9.

Koo et al. (2018) created other DMD mouse models, with these ones carrying frameshifting mutations at *Dmd* exon 23 [66]. Among the mutants generated, the authors looked further into those with a 14 bp deletion or 1 bp insertion in exon 23—both of which lacked dystrophin in examined TA samples by Western blot and immunostaining. Sarcolemmal neuronal nitric oxide synthase (nNOS) localization was also not observed. Muscle function was further evaluated in the 14 bp deletion model, and a significant decrease in specific force of the TA was observed. In the same study, both models were used to test the feasibility of employing CjCas9 (from *Campylobacter jejuni*) for DMD CRISPR therapy for the first time in vivo. Indeed, their findings supported the use of CjCas9 for DMD genome-editing therapy, which is beneficial for the field given that the CjCas9 cDNA has a considerably smaller size than that of SpCas9 [33,43]. This aids packaging into viral vectors, consequently facilitating treatment. On the other hand, Kim et al. (2017) developed a DMD mouse model using a cytidine deaminase fused to catalytically inactive Cas9, introducing a point mutation that leads to a premature stop codon in exon 20 of the *Dmd* gene [72]. While this model (called D108) has been used in a proof-of-principle study to demonstrate the feasibility of in vivo CRISPR base editing as a therapeutic approach for DMD [63], limited phenotypic analysis was performed and so we know very little about how well this model recapitulates the symptoms in patients. The model exhibits a lack of dystrophin and nNOS at the sarcolemma, however.

Humanized DMD mice are emerging models in the field, likely since the first transgenic mouse carrying a full-length, functional copy of the human *DMD* gene was created by ‘t Hoen et al. in 2008 [73]. Tissue-specific expression of the various *DMD* transcript isoforms in these mice was similar to that in humans. However, the expression levels of certain isoforms differed in these mice, e.g., the predominantly skeletal muscle-specific Dp427m isoform was expressed at twice the amount in the *hDMD* mice. The availability of this model enables testing of therapies that rely on the human *DMD* sequence, such as exon skipping [74] and genome editing [65,75]. A main limitation of this model though is that *hDMD* mice are phenotypically normal, and thus would not be useful for determining how well a given therapy restores dystrophin synthesis or improves muscle structure and function.

Fortunately, CRISPR can be used to edit these *hDMD* mice, making targeted mutations in the human *DMD* transgene to create a mutant transcript and cause dystrophin loss. Young et al. (2017) generated one such model, with *hDMD* mice carrying an out-of-frame exon 45 deletion (hDMD del45) [65]. When crossed onto the original *mdx* or *mdx*D2 (*mdx* DBA/2) background, dystrophin was absent in the skeletal muscles and heart by Western blot and immunostaining, save for a few, rare RFs. On the *mdx*D2 background, the skeletal muscles of hDMD del45 mice had observable dystrophic pathology: fibrosis, inflammatory infiltration, and calcium deposition. These mice can be used for testing therapies targeting the distal *DMD* mutation hotspot, such as exon 45–55 skipping, which Young et al. also demonstrated in their model as a proof of concept. Furthermore, we previously reported that an astounding 1554 of 4929 (31.5%) *DMD* deletion-carrying individuals in the Leiden Open Variation Database (LOVD) have deletions beginning at exon 45 [76], which emphasizes the clinical utility of the hDMD del45 model. Take note that there is another humanized DMD mouse model, del52hDMD/*mdx*, created by genome editing that also shows dystrophic phenotypes and can be used for therapeutic testing [75]. However, as this model was generated using transcription activator-like effector nuclease (TALEN) editing, we only briefly mention it here for reference.

### 3.2. Rat

In 2014, Nakamura et al. generated a number of inbred Wistar-Imamichi rats harboring different mutations in the *Dmd* gene using CRISPR, by injecting two gRNAs (one each targeting exons 3 and 16) and Cas9 mRNA into zygotes [57]. Nine out of ten F0 male progeny had detectable mutations in the *Dmd* gene, having deletions of 1–577 bp or insertions of 1–4 bp in one or both exons. More deletion than insertion events were observed overall, with some rats mosaic for mutations. All out-of-frame mutant males showed a lack of dystrophin in the TA by Western blotting or immunostaining. On the other hand, in-frame mutants had reduced dystrophin levels. F0 mutant rats had similar body weights to the wild-types throughout evaluation, up to 13 weeks. At 13 weeks of age, the TAs of mutant rats showed typical signs of dystrophic histopathology, e.g., degeneration, fibrosis, increased CNF counts, decreased myofiber size, and elevated fat levels, albeit with some individual variability owing to the non-isogenic nature of the colony. The diaphragm and heart also showed dystrophic signs but require further characterization. The F0 mutant rats displayed significantly reduced muscle function as per the wire hanging test. F1 mutant rats exhibited mostly similar phenotypes as the F0 rats at 4 weeks of age, including significantly elevated CK levels at approximately 4–5 times higher than wild-type levels.

The authors suggest that these dystrophin-deficient rats make for a more severe model of DMD than the *mdx* mice, particularly with regard to the heart [57]. Deeper investigation into the cardiac function of these rats is needed, as only histological observations were done. It is likely though that cardiac phenotypes will become apparent with age, as signs of diastolic dysfunction and structural remodeling of the left ventricle could be observed as early as 3 months of age in a different DMD rat model, one created by the introduction of mutations into *Dmd* exon 23 using TALEN genome editing [77]. Nakamura et al. also discuss that their DMD model rats exhibited more continuous disease progression than the *mdx* mice [57], which is more representative of what is observed in patients. Generally speaking, their larger body size and more widely recognized suitability to behavioral studies also provide certain advantages to using rats for therapeutic studies compared to mice [57,78,79]. However, results from the use of these DMD rats (or any dystrophin-deficient rat, for that matter) in the development of DMD therapies have yet to be reported.

### 3.3. Pig

The search for large animal models of DMD was partly initiated by the inability of rodent models to fully recapitulate patient phenotypes. A classic example would be the mild phenotype seen in *mdx* mice [22], as we have mentioned previously. There was also the issue of scale up, e.g., determining the human-appropriate dose, administration route, and treatment schedule for a given drug, which could be better managed with a more comparably sized animal model [80]. Use of canine DMD models, e.g., the Golden Retriever model of DMD (GRMD) [81] and Canine X-linked Muscular Dystrophy in Japan (CXMDJ) [28] dogs, emerged in response to these needs. While these dogs certainly exhibited more severe clinical signs compared to their rodent counterparts and provided us useful information on the pathogenesis of DMD, they displayed wide phenotypic variability across individuals that made pre-clinical study difficult [24,27,80]. Groups, therefore, looked into developing other large animal models of DMD.

As pigs share a high degree of similarity with the genetic, physiological, and anatomical characteristics of humans compared to mice or dogs, they soon became an attractive candidate for the development of a new DMD model [80,82,83]. In particular, the porcine heart functions almost identically to human hearts that parts of it are routinely used in cardiac surgery to replace patient valves and aortic roots [80]. This presents advantages in potentially modelling the cardiac aspects of DMD. A number of studies support the feasibility of using genome editing to create porcine disease models, with CRISPR/Cas9 reportedly being more efficient in generating mutants as opposed to zinc finger nucleases (ZFNs) or TALENs [84]. The first and only described attempt to develop a DMD pig model via CRISPR/Cas9 thus far was that by Yu et al. in 2016 [85]. Yu and colleagues microinjected Cas9 mRNA and a gRNA targeting *DMD* exon 27 into the cytoplasm of 1-cell stage embryos from Diannan miniature pigs, subsequently transferring the embryos into surrogate sows. Two piglets were successfully born out of the 98 injected embryos—only one of which had mutations in *DMD*—and both died shortly after birth.

The mutant pig died 52 days post-birth and carried four different indel mutations in the *DMD* gene that varied in distribution and level across tissues. The total efficiency of frame-disrupting mutations was 60% in skeletal muscle. Dystrophin levels in the mutant pig were lower than the age-matched wild-type in the biceps femoris, heart, and intestine, but not as pronounced. Typical dystrophic features such as reduced myofiber size, CNF presence, necrosis and myofiber disorganization were observed in skeletal muscles. The heart had seemingly hypertrophic foci. The severely shortened lifespan of the mutant pig generated by Yu et al. is similar to what was observed for the *DMD* exon 52-deleted pigs of Klymiuk et al. (2013) that mostly died within the first week of life [86]. As this drastically reduced survival prevents the use and efficient production of porcine DMD models for pre-clinical study, investigations into why this occurs or modifications in the genome-editing approach may help overcome the issue. The longer gestational period of pigs at 114 days, their requirement for larger living spaces and costly maintenance, as well as federal regulations limiting the use of genetically engineered pigs [24,80,87] are all challenges that have to be surmounted for using this DMD model in practice.

### 3.4. Rabbit

Compared to pigs, rabbits are more suitable in terms of productivity and cost while maintaining greater similarity at the genetic, physiological, and anatomical levels to humans than mice [24,88,89]. Rabbits have a gestational duration of 31 days, the capacity to breed throughout the year, and have reduced space requirements than dogs or pigs [24]. Their intermediate size also provides the advantages that come with increased body size without much of the financial burden [90]. Sui et al. (2018) recently developed a DMD rabbit model by co-injecting Cas9 mRNA and two gRNAs targeting *DMD* exon 51 into rabbit zygotes [89]. A total of 33 live rabbits were obtained out of 128 injected zygotes—26 of which had at least one mutated *DMD* allele. Indels of various sizes were detected, with some rabbits having more than one mutation along the same allele or in both alleles. The mutant rabbits had significantly lower *DMD* transcript levels, complete dystrophin loss as well as considerably reduced α-sarcoglycan and glycosylated α-dystroglycan at skeletal muscle membranes.

Significantly reduced mobility in the 1 hr walking (by more than 50% less than wild-type) and step-climbing tests were observed in the DMD rabbits, together with forelimb paralysis. Survival was decreased, with 42.6% of the rabbits dying by 20 weeks. In contrast to wild-type, 5-month-old mutant rabbit skeletal muscle showed apparent dystrophic phenotypes including a significantly greater proportion of CNFs, increased fibrosis, fat replacement, and significantly reduced fiber areas to less than half the average wild-type values. Four times higher serum CK levels were consistently observed in the mutants between 8 and 20 weeks of age. More importantly, unlike *mdx* mice that do not experience obvious cardiac dysfunction until 1 year of age [89,91], DMD rabbits show significantly reduced ejection fraction (EF) and fractional shortening (FS) compared to wild-type as early as 4 months old. Histological analysis of cardiac muscle revealed dystrophic symptoms, despite the lack of obvious differences in gross heart anatomy between mutant and healthy control rabbits. This positions *DMD*-mutant rabbits as being favorable models for studying the cardiac phenotype of DMD and for evaluating treatments directed at the dystrophic heart. The model is yet to be used for therapeutic testing.

### 3.5. Monkey

In the context of DMD therapy development, monkeys have mostly been used for pharmacological study, safety evaluation, and the testing of treatments that generally enhance muscle growth [92,93,94]. The lack of a suitable monkey model of DMD has prevented more thorough assessments of dystrophin-targeting therapies in these animals. The situation changed recently, however, with the generation of *DMD*-mutant rhesus monkeys (*Macaca mulatta*) by Chen et al. as reported in 2015 [95]. Chen and colleagues used CRISPR/Cas9 to introduce mutations in exons 4 and 46 of the monkey *DMD* gene. A total of 2 stillborn and 9 live monkeys carrying the desired mutations resulted from the editing process, where 179 embryos were injected. Mosaic mutations were reported in both stillborn and in some live monkeys. As both wild-type and mutant stillborn monkeys were observed, the authors did not attribute this condition to CRISPR/Cas9 activity.

Tissues of the stillborn monkeys (1 male, 1 female) were further analyzed, revealing that these contained 3 different frameshifting mutations in exon 4: a deletion of 2 bp, and insertions of 2 bp and 20 bp. Varying mutation rates of each type were observed across tissues and between monkeys, with 67% (male) or 87% (female) of *DMD* alleles in skeletal muscle mutated in total as evaluated by RT-PCR. Dystrophin reduction in muscle positively correlated with mutation load. Histological analysis of skeletal muscle samples (muscle unspecified) from the stillborn monkeys revealed a general decrease in fiber size, with the appearance of hypertrophic myofiber clusters, 12.5/17.5% CNFs, and increased interstitial space area. It is postulated that these dystrophic phenotypes began manifesting at an earlier time before birth. Phenotypic analysis was not performed for the live monkeys, which had a variety of frameshifting mutations in exons 4 and/or 46. In a succeeding study, the same group showed no off-target editing at known coding genomic regions in 2 of the live mutant monkeys via whole genome sequencing [96]. A total of five validated off-target indels were detected—four in intergenic regions, and one in an intron. No study on the use of these monkeys in therapeutic testing has been published as of present.

## 4. Conclusions

The use of CRISPR has expanded the DMD animal model repertoire, with the creation of at least 11 new models in total within a 6 year time period—8 of which were during the last 3 years (Table 2). This has allowed for better phenotypic and mutational representation in vivo, bringing the field closer to modelling the DMD patient population. Researchers are now presented with a wider selection of models to choose from for therapeutic studies, with the list of choices likely to grow in the coming years. The availability of such models also mobilizes efforts to further understand DMD pathology and progression. However, it is important to recognize that each model still has its own advantages and disadvantages (Table 3). For instance, while we can now test more personalized antisense or genome-editing therapies in mice, we still have not been able to faithfully recapitulate DMD patient cardiac phenotypes in this animal. And despite having the opportunity to achieve closer phenotypic representation in a larger, more “human-like” animal such as the pig, use of the model is hampered by its very early mortality [85,86]. Other issues surrounding the use of these models, particularly for the non-rodent ones, include cost, space, maintenance, government regulation, and public opinion [24]—all of which have to be duly considered prior to starting a therapeutic study.

Moving forward, a more in-depth investigation into the phenotypes of these new animal models is required to better determine their appropriateness for pre-clinical study. The fact that mosaics are being generated for some models [57,85,89,95] is concerning, as this will certainly influence the results of therapeutic testing. Developing methods to increase the uniformity of in vivo CRISPR-mediated mutagenesis will be helpful [97,98]; otherwise, careful analysis of mutation load and tissue-specific distribution is required in individual animals, perhaps even across generations. Finally, we should understand that each animal system inherently possesses certain fixed limitations (e.g., size, physiology, anatomy, etc.) and a model, by definition, cannot be expected to recapitulate all aspects of the DMD patient phenotype. Different models are useful for different purposes, and each must be used according to its strengths. One article suggests that we think of each model as representing different stages of DMD progression—for example, with murine models coinciding with the earliest phase of the disease, canine models with presentation during 5–10 years of age, and the rabbit model with the later phases associated with cardiac symptoms [24]. Therapies have to be tested depending on their desired clinical endpoint, which can only be accurately evaluated with the right animal model. The future of DMD therapy development will therefore likely require a holistic approach, using a combination of in vivo and in vitro models according to their respective strengths for the most accurate evaluation of a candidate drug.

## Figures and Tables

**Table 1:** A list of selected Cas9 enzymes and their characteristics.

Cas9 Enzyme	Source Organism	PAM Site (5′-3′)	Size (cDNA, Protein)	Properties	Ref/s
SpCas9	*Streptococcus pyogenes*	NGG	4.10 kbp, 1,368 aa	Most widely used Cas9; common PAM site, but also more susceptible to off-target effects, multiple variants available	[30,38]
SaCas9	*Staphylococcus* *aureus*	NNGRRT	3.16 kbp, 1,053 aa	Small Cas9 with high specificity and efficiency	[39]
BlatCas9	*Brevibacillus* *laterosporus*	NNNNCNDD	3.28 kbp, 1,092 aa	Small Cas9 with broad targeting range	[40]
CdCas9	*Corynebacterium diphtheriae*	NNRHHHY	3.25 kbp, 1,084 aa	Diverse PAM sequence recognition, more robust DNA cleavage activity compared to traditional Cas9 orthologues	[41]
CjCas9	*Campylobacter jejuni*	NNNNACA	2.95 kbp, 984 aa	Smallest available Cas9, lack of a protospacer flanking sequence allows for greater target flexibility, shown to target RNA as well	[42,43]
FnCas9	*Francisella novicida*	NGG	4.89 kbp, 1,629 aa	High binding specificity and low off-target effect compared to SpCas9 while having similar PAM site, size may deter delivery	[44]
GeoCas9	*Geobacillus stearothermophilus*	CRAA	3.26 kbp, 1,087 aa	Small thermotolerant Cas9 active in temperatures up to 70 °C, shown to have improved ribonucleoprotein delivery	[45]
NmCas9	*Neisseria meningitidis*	NNNNGATT	3.25 kbp, 1,082 aa	Small Cas9 with high specificity, lower off-target effect compared to SpCas9	[46,47]
ScCas9	*Streptococcus canis*	NNG	4.161 kbp, 1,386 aa	Homologous protein to SpCas9 with higher levels of specificity, size may deter delivery	[48]
St1Cas9	*Streptococcus thermophilus*	NNAGAAW	3.36 kbp, 1,121 aa	Small Cas9 that can induce error-prone NHEJ-mediated targeted mutagenesis as efficiently as SpCas9	[49]

Abbreviations: PAM, protospacer adjacent motif; NHEJ, non-homologous end joining.

**Table 2:** Characteristics of the various CRISPR-generated Duchenne muscular dystrophy (DMD) animal models discussed in this review.

Animal	Study	Strain/Breed	Dystrophin Mutation	Features (In Males, Unless Specified)	Therapies Tested
Mouse	Kim et al. (2017)	C57BL6/J	*Dmd* ex20 point mutation (Q871Stop)	Dystrophin and nNOS absent in skeletal muscle sarcolemma	CRISPR editing of ex20 point mutation [63]
	Amoasii et al. (2017)	C57BL6/J	*Dmd* ex50 deletion	Dystrophin absent in skeletal and cardiac muscles, histopathology at 3 weeks, decreased forelimb grip strength at 2 months	CRISPR reframing or skipping of ex51 [64]
	Young et al. (2017)	C57BL/10, DBA/2	*DMD* ex45 deletion	Humanized model, dystrophin absent in skeletal and cardiac muscles on *mdx* and *mdx*D2 backgrounds, histopathology at 6 weeks on *mdx*D2 background	CRISPR deletion of ex45-55 [65]
	Koo et al. (2018)	C57BL6/J	various *Dmd* exon 23 indel mutations (e.g., −14bp, +1bp)	Dystrophin absent in skeletal muscles, nNOS absent in skeletal muscle sarcolemma, decreased TA-specific force at nearly 4 months	CRISPR reframing of ex23 [66]
	Min et al. (2019)	C57BL6/J	*Dmd* ex44 deletion	Dystrophin absent in skeletal and cardiac muscles, histopathology at 4 weeks, decreased EDL-specific force at 4 weeks and forelimb grip strength at 8 weeks	CRISPR reframing or skipping of ex51/53 [67] or of ex45 [68]
	Egorova et al. (2019)	C57BL6/J × CBA	*Dmd* ex8-34 deletion	Dystrophin, DAGC members absent in skeletal muscles, histopathology at 12 weeks, decreased TA force parameters and wire hanging test performance across age (2–12 months)	None
	Amoasii et al. (2019)	Not indicated	*Dmd* ex50 deletion	Similar to those from Amoasii et al. (2017), with capability of in vivo non-invasive monitoring of dystrophin levels via luciferase expression	CRISPR reframing or skipping of ex51 [69]
Rat	Nakamura et al. (2014)	Wistar-Imamichi	various *Dmd* exon 3 and/or 16 indel mutations	Dystrophin absent or reduced in skeletal muscle, histopathology at approximately 4 or 13 weeks for skeletal muscle and at 13 weeks for the heart, decreased wire hanging test performance	None
Pig	Yu et al. (2016)	Diannan miniature pig	various *DMD* exon 27 indel mutations	Only one mutant obtained with unspecified sex and very early mortality at 52 days post-birth, dystrophin reduced in skeletal and cardiac muscles, histopathology observed at autopsy with the heart having enlarged, discolored foci	Not applicable
Rabbit	Sui et al. (2018)	New Zealand	various *DMD* exon 51 indel mutations	Sex unspecified: reduced survival mostly by 20 weeks, dystrophin absent in skeletal and presumably cardiac muscles, histopathology at 5 months, reduced mobility at 2–3 months, decreased systolic function at 4 months	None
Monkey	Chen et al. (2015)	Rhesus	various *DMD* exon 4 and/or 46 indel mutations	Only stillborn monkeys analyzed (male and female): dystrophin reduced in muscle, histopathology observed	None

Abbreviations: ex, exon; nNOS, neuronal nitric oxide synthase; TA, tibialis anterior; EDL, extensor digitorum longus.

**Table 3:** Advantages and disadvantages of the CRISPR-generated DMD animal models.

Model	Advantages	Disadvantages
Mouse	Wide number of mutations, humanized model available, easy to breed and handle, good for powered studies, relatively inexpensive	Mild phenotype overall, cardiac phenotypes require further investigation, small body size
Rat	Potential cardiac phenotype, continuous DMD progression, suitable for behavioral studies, easy to breed and handle, good for powered studies, relatively inexpensive	Non-isogenic colony used for mutant generation, mosaic mutations require careful consideration, small body size
Pig	Good genetic, physiological, and anatomical similarity to humans, large body size	Very early mortality prevents breeding and further study, long gestational period, expensive to maintain and house
Rabbit	Good genetic, physiological, and anatomical similarity to humans, strong cardiac phenotype, intermediate body size, relatively short gestational period	May be expensive to maintain and house (but less so than the pig or monkey), mosaic mutations require careful consideration
Monkey	Least evolutionary distance and hence greatest similarity to humans, large body size	Lack of phenotypic analysis for living mutants, long gestational period, mosaic mutations require careful consideration, expensive to maintain and house
